# Using structured problem solving to promote fluid consumption in the prevention of urinary stones with hydration (PUSH) trial

**DOI:** 10.1186/s12882-024-03605-y

**Published:** 2024-05-28

**Authors:** Peter P. Reese, Salima Shah, Emily Funsten, Sandra Amaral, Janet Audrain-McGovern, Kristen Koepsell, Hunter Wessells, Jonathan D. Harper, Rebecca McCune, Charles D. Scales, Ziya Kirkali, Naim M. Maalouf, H. Henry Lai, Alana C. Desai, Hussein R. Al-Khalidi, Gregory E. Tasian

**Affiliations:** 1 Renal-Electrolyte and Hypertension Division, Department of Medicine, Perelman School of Medicine, University of Pennsylvania, Philadelphia, PA USA; 2 Division of Urology, Children’s Hospital of Philadelphia, Philadelphia, PA USA; 3 University of New Mexico School of Medicine, Albuquerque, NM USA; 4 Department of Pediatrics, Children’s Hospital of Philadelphia, Philadelphia, PA USA; 5 Department of Psychiatry, Perelman School of Medicine, Philadelphia, PA USA; 6 Bristol Myers Squibb, Princeton, NJ USA; 7 Department of Urology, University of Washington, Seattle, WA USA; 8 Department of Population Health Sciences, Duke University School of Medicine, Durham, NC USA; 9 Department of Surgery (Urology), Duke Surgical Center for Outcomes Research & Equity in Surgery, Duke University School of Medicine, Durham, NC USA; 10 Duke Clinical Research Institute, Duke University School of Medicine, Durham, NC USA; 11 National Institute of Diabetes and Digestive and Kidney Diseases, Bethesda, MD USA; 12 Department of Internal Medicine and Charles and Jane Pak Center for Mineral Metabolism and Clinical Research, University of Texas Southwestern Medical Center, Dallas, TX USA; 13 Department of Surgery (Urology), Washington University in St. Louis, St. Louis, MO USA; 14 Department of Anesthesiology, Washington University in St. Louis, St. Louis, MO USA; 15 Department of Biostatistics & Bioinformatics, Duke University School of Medicine, Durham, NC USA; 16 Center for Clinical Epidemiology and Biostatistics, University of Pennsylvania, 917 Blockley Hall | 423 Guardian Drive, Philadelphia, PA 19104 USA

**Keywords:** Kidney stone, Behavior change, Financial incentives

## Abstract

**Background:**

Structured Problem Solving (SPS) is a patient-centered approach to promoting behavior change that relies on productive collaboration between coaches and participants and reinforces participant autonomy. We aimed to describe the design, implementation, and assessment of SPS in the multicenter Prevention of Urinary Stones with Hydration (PUSH) randomized trial.

**Methods:**

In the PUSH trial, individuals with a history of urinary stone disease and low urine output were randomized to control versus a multicomponent intervention including SPS that was designed to promote fluid consumption and thereby prevent recurrent stones. We provide details specifically about training and fidelity assessment of the SPS coaches. We report on implementation experiences related to SPS during the initial conduct of the trial.

**Results:**

With training and fidelity assessment, coaches in the PUSH trial applied SPS to help participants overcome barriers to fluid consumption. In some cases, coaches faced implementation barriers such as variable participant engagement that required tailoring their work with specific participants. The coaches also faced challenges including balancing rapport with problem solving, and role clarity for the coaches.

**Conclusions:**

We adapted SPS to the setting of kidney stone prevention and overcame challenges in implementation, such as variable patient engagement. Tools from the PUSH trial may be useful to apply to other health behavior change settings in nephrology and other areas of clinical care.

**Trial registration:**

ClinicalTrials.gov Identifier NCT03244189.

**Supplementary Information:**

The online version contains supplementary material available at 10.1186/s12882-024-03605-y.

## Background

Urinary stone disease (USD) is a common and painful problem. In 2018, 11% of Americans suffered from USD [1]. Individuals typically experience severe discomfort during stone passage and have an increased risk for chronic kidney disease [2], bone fracture [3, 4], hypertension [5–8], and cardiovascular disease [4, 9, 10]. The probability of a recurrent symptomatic stone event is 50% within 3 to 10 years of initial diagnosis [11–15]. Strong evidence suggests that high fluid consumption will reduce USD recurrence [16]. However, many individuals with stones are unable to consistently increase their fluid consumption due to diverse reasons such as forgetting, abdominal symptoms, bladder irritability, lack of access to a bathroom, or lack of conviction that the fluids will prevent stones.

Structured Problem Solving (SPS) is a promising approach to promoting health behavior change that has not been tested in the setting of USD prevention. SPS has been successfully applied to improve healthy behaviors in other settings such as HIV medication adherence and diabetes self-management [17–19]. Rooted in Problem Solving Therapy, SPS complements the positive effects of motivational interviewing [20–23] by identifying pragmatic solutions to behavior change at the level of an individual. SPS is utilized to improve adherence to positive health behaviors or to cease harmful behaviors, either as a stand-alone intervention or as part of a multicomponent intervention. In practice, SPS is often facilitated by a “health coach,” an individual who partners with the participant to elicit, develop, and implement solutions to changing the health behavior. This approach to improving health behaviors is intended to address ambivalence to change by helping patients (a) identify personal barriers to change, (b) develop feasible solutions to the problem that they are able to implement, and (c) evaluate the effectiveness of the approach and refine it over time as needed [24]. Similar to some other behavior change strategies, SPS should support patient autonomy by helping the patient’s behavior align with their goals [25]. Notably, SPS is not a form of psychotherapy, but rather a pragmatic and customized approach to improving behaviors including those related to health.

In the intervention designed for the Prevention of Urinary Stones with Hydration (PUSH) randomized trial, investigators in the Urinary Stone Disease Research Network identified SPS as a behavioral intervention complementary to financial incentives. The overall goal of PUSH was to determine whether a multicomponent behavioral intervention to increase and maintain high fluid consumption would reduce the risk of recurrent urinary stones. We previously described the overall trial design [26]. In the PUSH intervention arm, financial incentives for meeting prescribed fluid consumption goals were intended to help participants focus on drinking fluids, while SPS was implemented to help participants develop personalized solutions to overcome barriers to increasing their overall fluid consumption. In designing the PUSH study, we reviewed existing literature and found that some published studies included insufficient detail to replicate key elements of the intervention, including training of the coaches, the approach used to elicit barriers and solutions to the health behavior, and fidelity assessment for the work of the coaches [27, 28]. In addition, SPS has not been customized for the USD clinical context, in which fluid consumption is a major component of stone prevention. We developed approaches and materials appropriate to the USD setting that were implemented at the 6 PUSH clinical centers and monitored by the data coordinating center. The aim of the present manuscript is to focus specifically on describing the design, implementation, and assessment of SPS in the PUSH randomized trial [26].

## Methods

### Methods: overview of the PUSH trial

PUSH is a two-arm randomized controlled trial that enrolled 1,658 individuals who consented to participate for 24 months (ClinicalTrials.gov NCT03244189). Adults and adolescents aged 12 years or above with a symptomatic urinary stone within the past 3 years (or 5 years if asymptomatic stone recurrence had occurred by imaging), low urine volume demonstrated on a 24-hour urine collection, and access to a smart phone or tablet were eligible. The primary endpoint was a recurrent urinary stone event, defined as symptomatic passage of a urinary stone or a procedure to remove a stone within 2 years of randomization. All participants received a smart Bluetooth-enabled water bottle (Hidrate Spark) that automatically recorded fluid consumption and transferred data to a phone or tablet. All participants completed 24-hour urine collections at baseline and every 6 months [26].

**Fig. 1:**
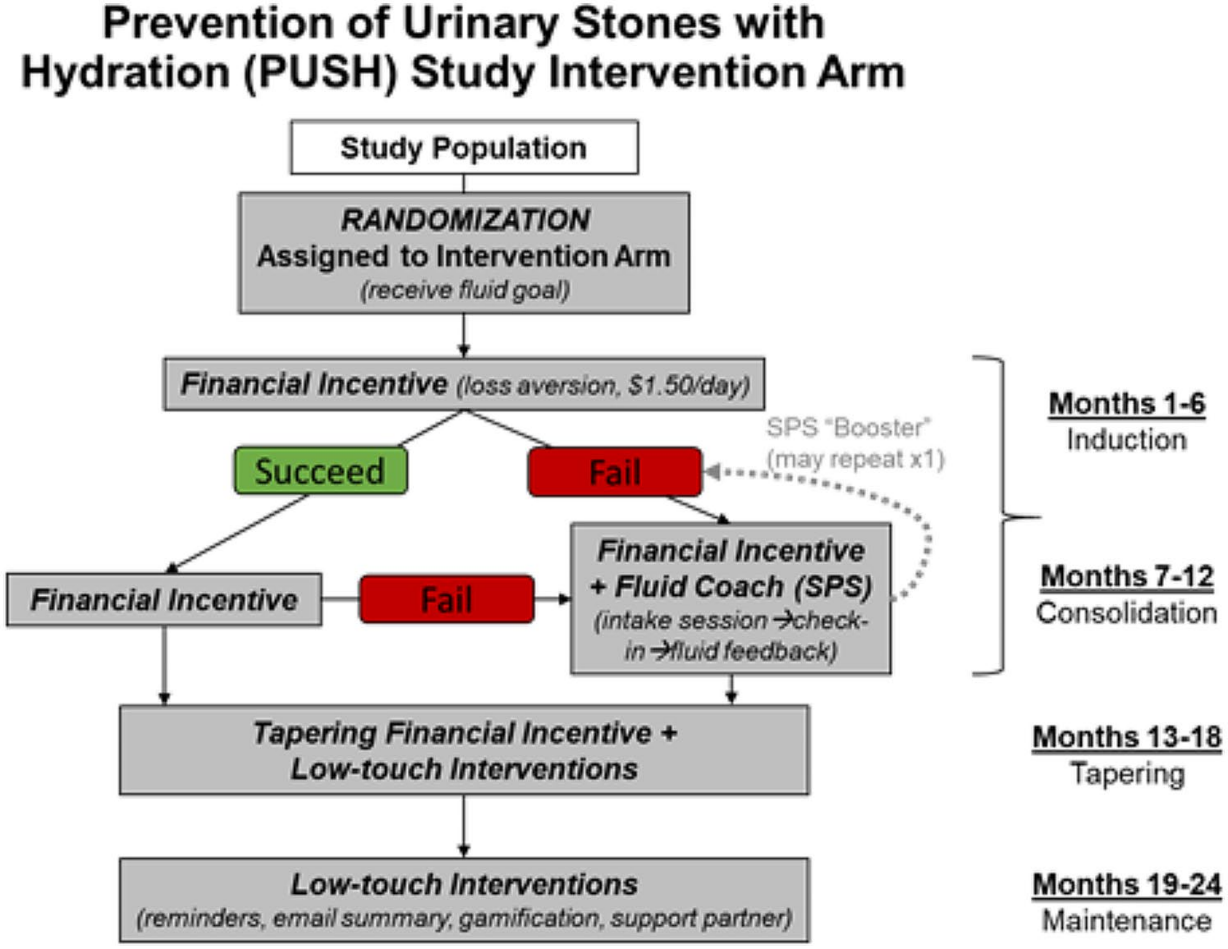
Prevention of Urinary Stones with Hydration (PUSH) Study Intervention Arm

Figure 1 provides a schematic of the study intervention’s components. Briefly, participants randomized to the intervention arm received a personalized fluid prescription for additional fluid they should consume each day to avoid stone recurrences. For adults, the goal was to drink sufficient fluids to produce ≥ 2.5 L of urine/day. If intervention arm recipients met or exceeded that prescribed additional fluid volume using the smart bottle, then they were eligible for a financial incentive of $1.50/day, which was tapered during months 12–18.

During the first 12 months of the trial, if fluid consumption goals were not met on > 80% of days during two 14-day assessment periods, intervention arm participants qualified for SPS. SPS consisted of an intake session with a PUSH health coach in which participants completed a six-step process that elicited barriers to maintaining high fluid consumption, solutions to overcome them, and prioritized the most feasible solutions. This was followed by a 1-week check-in and then monthly communications with feedback about fluid consumption. If participants continued to not meet daily fluid consumption goals, they received up to two “booster” coaching sessions that repeated the six SPS steps.

After either completing coaching or at the end of the first 12 months, intervention arm participants were offered automated “low touch” supports to maintain high fluid consumption, such as having fluid consumption data sent to a self-chosen “adherence support partner.” The control arm participants neither received financial incentives nor SPS. Information about the overall trial design has been published previously, but did not include details about the SPS intervention [26]. This manuscript focuses on the SPS component of the intervention.

### Patient and public involvement

The protocol was prepared by investigators experienced in treating patients with USD. Six USD patients and 2 parents of adolescent patients — a total of 8 adult individuals from the geographic locations of the clinical centers, Scientific Data Research Center (Durham, North Carolina), and the Washington, DC area — were involved during the protocol development process. All patients and parents were interviewed either by phone or provided email feedback prior to the final design of the study protocol. One urologist conducted the semi-structured interviews by directly asking open-ended questions related to: (a) the importance of the research question, (b) patients’ experience in relevance to the scientific question that would be investigated, (c) the burden of study procedures and acceptability of the intervention by the patients, and (d) any suggestions to improve the quality of the protocol.

Interim periodic electronic newsletters were sent to the participants throughout the clinical trial. At the time of this writing, recruitment of study participants is complete and follow-up is in progress. Once the analyses of the data are complete, the results will be shared with the study participants. The participants will also have access to any secondary papers derived from the study. The patients and parents who have helped in the development of the study protocol will be anonymously thanked in the primary outcome paper(s).

### Application of SPS in the PUSH trial

#### Hiring and training

SPS coaches were hired at each clinical center. Where possible, sites hired individuals with a background or experience in counseling patients about behavioral health, such as social work training. The rationale for employing coaches in the same geographic location was that a coach in Dallas, Texas (one site) might be better prepared to discuss local issues such as a heat wave, traffic patterns, or a recent high-profile sporting event with a Dallas-based participant versus a coach located in Seattle, Washington (a different site). Being in the same time zone also facilitated scheduling coaching sessions.

Two investigators with a background in behavior change theory and behavioral interventions (S.A., J.A.-M.) developed and carried out SPS training. At the start of the trial, the training consisted of an in-person retreat, followed by additional supervision and fidelity assessment over several months. The training reviewed general information about the biology and treatment of USD and then introduced coaches to general concepts about successful behavior change and the implementation of SPS. The didactic sessions reviewed common misconceptions about USD, such as the idea that drinking more fluids may cause a stone to be dislodged and passed in the urine or that coffee promotes stones. The training reviewed the importance of building rapport, active listening, asking nonjudgmental questions, maintaining a supportive and empathetic tone, and setting boundaries where necessary. Next, the training covered a step-by-step approach to identifying the participant’s specific barriers to greater fluid consumption and mutually identifying feasible solutions (see Appendix 1 in Additional File 1). The coaches were trained to review participant fluid adherence data and focus on days when participants were unable to achieve the recommended volume of fluid consumption using the study-provided water bottle. Participants were then encouraged to come up with solutions to overcome fluid consumption barriers. The goal was to brainstorm as many solutions as possible. Coaches were encouraged to be systematic about helping patients identify different types of barriers, such as work demands, lack of access to bathrooms, physical feelings of satiation, not having fluids handy, or forgetting to consume fluids on hand. The solutions might involve substantial lifestyle changes (such as workplace accommodations to have better bathroom access) or small steps (such as flavoring water with lemon).

To improve coaches’ understanding of patients’ lived experience, the coaching trainers presented a series of hypothetical phenotypes of participants (10 adult and 4 adolescent) with diverse personalities, personal circumstances, and reasons for insufficient fluid consumption. During training, which included role playing, coaches learned how to engage participants and apply SPS in the setting of subsequent “booster” meetings. Coaches were required to achieve specific training milestones before being assigned trial participants. Appendix 2 provides sections from the Manual of Procedures used by coaches, with detailed information about training. Appendix 3 provides examples of hypothetical stone patients that the coaches discussed.

#### Fidelity assessment and longitudinal support for coaches

A fidelity assessment was completed after the first week of coaching. Each SPS coach audio-recorded one or more of the initial intake meetings with a participant, and recordings were evaluated by a coaching supervisor. A booster meeting was also reviewed. Appendix 4 describes elements of fidelity assessment.

#### Challenges and solutions for implementing the SPS intervention

To provide insight into SPS engagement, we obtained data on all PUSH participants randomized to the intervention arm who qualified for SPS. We supplemented these data with insights from the coaches and their supervisors by reviewing the minutes of the SPS coaches’ meetings, which took place once or twice monthly. We also sent a set of structured questions to current coaches and evaluated written replies. Two co-authors (K.K. and S.S.) were also coaches.

## Results

Table 1 shows the characteristics of the 275 individuals (approximately 1/3 of participants assigned to the trial intervention arm) who qualified for SPS. These 275 individuals had not met fluid consumption goals despite receiving financial incentives. Among the 275, 147 (53%) completed their initial intake meeting with their health coach to assess their barriers to drinking more fluids. The mean age of patients who completed the SPS intake was 41 years. 65% were female. A total of 85.7% self-identified as White race, 10.2% as Black race, 2% as Other race, 1.4% as Asian race, and 0.7% as Multi-racial. A total of 37.4% reported having had one urinary stone event, while 62.6% had experienced recurrent urinary stones. The initial intake SPS meeting lasted a mean of 54 min (SD 16 min).

**Table 1:** Baseline demographic and clinical characteristics of intervention arm participants who qualified for structured problem solving

Demographics	Completed SPS (*n* = 147)	Declined SPS (*n* = 36)	Disengaged SPS (*n* = 92)
**Age in years median (25th − 75th percentiles), n**	41 (25–54), 147	40 (28–60), 36	40 (30–57), 92
**Female sex (n/N (%))**	95 / 147 (64.6%)	24 / 36 (66.7%)	49 / 92 (53.3%)
**Male sex (n/N (%))**	52 / 147 (35.4%)	12 / 36 (33.3%)	43 / 92 (46.7%)
Race (n/N (%))*			
White	126 / 147 (85.7%)	32 / 36 (88.9%)	82 / 92 (89.1%)
Black or African American	15 / 147 (10.2%)	2 / 36 (5.6%)	8 / 92 (8.7%)
American Indian or Alaska Native	0 / 147 (0.0%)	0 / 36 (0.0%)	0 / 92 (0.0%)
Asian	2 / 147 (1.4%)	0 / 36 (0.0%)	0 / 92 (0.0%)
Native Hawaiian or Other Pacific Islander	0 / 147 (0.0%)	0 / 36 (0.0%)	1 / 92 (1.1%)
Other	3 / 147 (2.0%)	2 / 36 (5.6%)	0 / 92 (0.0%)
Unknown	0 / 147 (0.0%)	0 / 36 (0.0%)	0 / 92 (0.0%)
Multiracial	1 / 147 (0.7%)	0 / 36 (0.0%)	1 / 92 (1.1%)
**Ethnicity (n/N (%))**			
Not Hispanic or Latino	132 / 147 (89.8%)	33 / 36 (91.7%)	81 / 92 (88.0%)
Hispanic or Latino	10 / 147 (6.8%)	1 / 36 (2.8%)	7 / 92 (7.6%)
Not reported	5 / 147 (3.4%)	1 / 36 (2.8%)	3 / 92 (3.3%)
Unknown	0 / 147 (0.0%)	1 / 36 (2.8%)	1 / 92 (1.1%)
**Household Income (n/N (%))**			
Less than $90,000	59 / 147 (40.1%)	17 / 36 (47.2%)	35 / 92 (38.0%)
$90,000 or more	60 / 147 (40.8%)	17 / 36 (47.2%)	39 / 92 (42.4%)
Other*	28 / 147 (19.0%)	2 / 36 (5.6%)	18 / 92 (19.6%)
**Employment Status (n/N (%))**			
Working full-time	82 / 147 (55.8%)	19 / 36 (52.8%)	56 / 92 (60.9%)
Working part-time	10 / 147 (6.8%)	2 / 36 (5.6%)	2 / 92 (2.2%)
Unemployed/looking for work	5 / 147 (3.4%)	0 / 36 (0.0%)	2 / 92 (2.2%)
Stay at home full time	6 / 147 (4.1%)	2 / 36 (5.6%)	5 / 92 (5.4%)
Retired	11 / 147 (7.5%)	5 / 36 (13.9%)	8 / 92 (8.7%)
Temporarily laid off/sick leave	1 / 147 (0.7%)	0 / 36 (0.0%)	2 / 92 (2.2%)
Permanently disabled	3 / 147 (2.0%)	0 / 36 (0.0%)	2 / 92 (2.2%)
Student	29 / 147 (19.7%)	8 / 36 (22.2%)	13 / 92 (14.1%)
Other**	0 / 147 (0.0%)	0 / 36 (0.0%)	2 / 92 (2.2%)
**Highest Education Level (n/N (%))**			
Less than high school degree	22 / 147 (15.0%)	5 / 36 (13.9%)	12 / 92 (13.0%)
High school graduate	8 / 147 (5.4%)	4 / 36 (11.1%)	9 / 92 (9.8%)
Some college or associates degree	30 / 147 (20.4%)	18 / 36 (50.0%)	21 / 92 (22.8%)
Bachelor’s degree	45 / 147 (30.6%)	2 / 36 (5.6%)	27 / 92 (29.3%)
Master’s or higher professional degree	39 / 147 (26.5%)	7 / 36 (19.4%)	21 / 92 (22.8%)
Prefer not to answer	3 / 147 (2.0%)	0 / 36 (0.0%)	2 / 92 (2.2%)
**Primary Health Care Insurance (n/N (%))**			
Private	122 / 147 (83.0%)	23 / 36 (63.9%)	70 / 92 (76.1%)
Medicare	11 / 147 (7.5%)	5 / 36 (13.9%)	11 / 92 (12.0%)
Medicaid	10 / 147 (6.8%)	2 / 36 (5.6%)	6 / 92 (6.5%)
Military Health Care	0 / 147 (0.0%)	4 / 36 (11.1%)	2 / 92 (2.2%)
State Specific	1 / 147 (0.7%)	0 / 36 (0.0%)	1 / 92 (1.1%)
Indian Health Service	0 / 147 (0.0%)	0 / 36 (0.0%)	0 / 92 (0.0%)
Uninsured	2 / 147 (1.4%)	1 / 36 (2.8%)	2 / 92 (2.2%)
Other**	1 / 147 (0.7%)	1 / 36 (2.8%)	0 / 92 (0.0%)
**Body Composition, median (25th − 75th percentiles), n**			
Weight (lbs)	170.0 (141.0–198.0), 147	191.5 (154.2–248.0), 36	176.0 (150.0–206.5), 92
Height (in)	66.00 (63.74–68.00), 146	66.00 (63.00–70.00), 35	66.69 (64.00–70.00), 92
Body mass index (kg/m^2^)	27.28 (23.72–31.35), 146	29.56 (26.57–37.51), 35	27.91 (23.20–31.97), 92
**Kidney stone occurrence (n/N (%))**			
First stone	55/147 (37.4%)	14/36 (38.9%)	37/92 (40.2%)
Recurrent stone	92/147 (62.6%)	22/36 (61.1%)	55/92 (59.8%)
**Diabetes mellitus (n/N (%))**	8 / 147 (5.4%)	5 / 36 (13.9%)	10 / 92 (10.9%)
**Hypertension (n/N (%))**	21 / 147 (14.3%)	3 / 36 (8.3%)	18 / 92 (19.6%)
**Kidney disease - not stone related (n/N (%))**	2 / 147 (1.4%)	0 / 36 (0.0%)	1 / 92 (1.1%)
**Myocardial infarction (n/N (%))**	1 / 147 (0.7%)	2 / 36 (5.6%)	1 / 92 (1.1%)
**Obesity (n/N (%))**	13 / 147 (8.8%)	6 / 36 (16.7%)	8 / 92 (8.7%)
**Study follow-up time in days, median (25th − 75th percentiles), n**	731 (660–768), 147	713 (418–740), 36	733 (651, 763), 92

* Includes don’t know and prefer not to answer

** Includes other and prefer not to answer

Among the 147 intervention arm participants who completed their SPS intake, the median number of coaching meetings was 4 (interquartile range: 2, 7). A total of 79 qualified for a “Booster” of SPS in which the health coach carried out a new intake meeting to review barriers to fluid consumption and to develop new solutions to overcome the barriers.

Among those who never completed the meeting with the health coach, a total of 36 explicitly refused to participate. We categorized the reasons provided by participants for refusing SPS into 4 broad groups: (1) Participant did not think that SPS would be helpful to improve fluid consumption and/or thinks fluid consumption will improve in the future without SPS; (2) Participant too busy to work with coach; (3) Participant believes that he or she qualified for SPS erroneously because of problems with the function of the study water bottle, rather than a true fluid consumption problem; and (4) Participant had a transient health issue (such as COVID) that temporarily interfered with fluid consumption, but then resolved.

The remaining 92 individuals who never completed the SPS intake were categorized as “disengaged,” meaning that they neither responded to attempts to schedule an intake nor provided a specific reason for not meeting a health coach. Table 1 shows that disengaged participants were 46.7% male, versus 35.3% of individuals who completed SPS and 33.3% who declined SPS, but otherwise, disengaged participants did not show large differences from the other two groups.

For the 147 participants who did engage in SPS, the coaches identified various challenges in helping these participants to improve fluid consumption. Table 2 enumerates these challenges, which are further described below.

**Table 2:** PUSH study challenges, consequences, and solutions

Challenge	Example	Consequences	Solution
Variable patient engagement	• Some participants who qualified for SPS did not respond to contacts or attend scheduled meetings • Adolescents often had challenges of busy schedules of school and after-school activities • For adolescents, engagement with parents/guardian could in some cases undermine rapport with coaches	• These participants could not benefit from SPS	• Repeated, respectful attempts to schedule meetings • Coaches to have flexibility in their schedules to accommodate participants • Coaches tried when possible to engage directly with adolescent participants or work to keep parental interactions from interfering with adolescent’s study participation • Even when participants refused SPS, study staff emphasized the value of remaining in the trial and enabling outcome ascertainment
Balancing rapport with focusing on problem solving	• Some participants were talkative during SPS encounters; topics might be tangential to the problem of kidney stones	• Coaches might build rapport through conversation but not make progress in helping participants overcome barriers to fluid intake • Coaches were trained to elicit strategies to improve fluid intake from participants, but sometimes had a tendency to volunteer their own solutions	• Coaches became skilled at friendly re-direction to keep focused on PUSH • Coaches tried to ask open-ended questions and encourage brainstorming about ways to improve fluid intake • Coaches first wait for participants to suggest their own solutions
Maintaining role clarity between SPS coaches versus study coordinators	• Participants often wanted coaches to help with solving technological problems related to wireless water bottles or financial incentives • Participants might report their potential kidney stone events to coaches, which might create ascertainment bias since control arm participants did not interact as often with study staff	• Participants might become frustrated with coaches or feel their needs were not met efficiently	• Coaches tried where possible to offer advice about bottle use, direct participants to online resources or refer the problem to a coordinator, and then redirect toward SPS
Study-specific barriers	• Wireless bottles sometimes did not transfer fluid intake data accurately • Documentation burden because coaches needed to toggle between several databases and record details of interactions	• Coaches might not be able to ascertain success of SPS • Coaches had less time to focus on SPS implementation	• Coaches tried to integrate fluid intake data with information provided verbally by the participant about fluid intake • Coaches tried to become more facile with study database use

### Implementation challenge 1: variable participant engagement

Coaches and participants had the option of meeting in person, but the evaluations and subsequent meetings were almost always carried out by phone or video connection, a very useful option once the COVID-19 pandemic started [29]. One challenge was some participants’ nonresponse to requests for the initial SPS meeting.

The coaches contacted non-responsive participants with respectful, repeated attempts to schedule a meeting while trying to maximize flexibility about times to accommodate participant needs. The manual of operations provided specific guidance about varying the means of outreach between email messages, text messages, and phone calls. The coaches balanced this approach with the need to maintain professional boundaries, for instance, by using an institution-designated phone or a Google phone number instead of their personal phone.

Adolescent participants presented certain challenges. Reaching adolescents during the school day (when most coaches were working) was often not feasible, because many adolescents had restricted access to their devices. For many adolescents, SPS had to accommodate demanding after-school activities. The coaches also contended with the frequent need to tactfully engage with parents or guardians. In some cases, the parent or guardian was more motivated to schedule the SPS than the study participant. The coaches reported that engaging with parents or guardians could in some cases facilitate scheduling SPS sessions, but in other cases could alienate the adolescent who resented parental involvement.

Some participants would respond to coaches’ messages but did not want to complete SPS sessions. The investigators emphasized the important dual goals of encouraging participation in SPS while strongly encouraging participants to remain in the trial to enable ascertainment of the outcome of kidney stones and related procedures. As a result, the coaches and coordinators formally designated some intervention arm participants as having opted out of SPS, and the coaches made no further attempts to contact them.

### Implementation challenge 2: balancing rapport with focus on problem solving

The coaches noted that some participants were talkative on a range of topics but did not seem oriented toward discussing fluid consumption. The coaches recognized the importance of building rapport with participants and being an active listener, but also staying focused on the goal of health improvement. Therefore, an important skill that coaches developed was redirection of the conversation toward stones and robust fluid consumption. In SPS implementation, some coaches noted their own tendencies to reflexively come up with their solutions for participants’ challenges. In discussion with the coaching supervisor and other coaches, the coaches recognized the better approach of asking open-ended questions and helping participants offer their own answers about how to integrate more fluid consumption into their lifestyles.

### Implementation challenge 3: role clarity

The role of the PUSH trial coaches was to engage eligible participants in SPS and help them implement solutions for fluid consumption. To avoid bias in outcome ascertainment between the intervention arm and control arm, the coaches’ role did not include asking about stones or evaluating the clinical information. If participants mentioned a kidney stone event, they were referred to the research coordinator and reminded to report the stone on the routine questionnaires about clinical outcomes that were sent to participants at regular intervals.

The coaches’ role did not encompass support of the smart water bottles or delivery of the financial incentives. Keeping these responsibilities separate was challenging at times for coaches. Because the smart water bottles sometimes were lost or malfunctioned (e.g., due to dead batteries or sensor problems), participants often wanted these problems addressed by their coaches. Indeed, it was necessary for coaches to ascertain whether a referral for SPS was due to a valid problem of insufficient fluid consumption or to erroneous data on fluid consumption related to lack of a functioning bottle. In these situations, the coaches asked the assigned study to resolve issues about payment for study activities.

### Implementation challenge 4: study-specific barriers

The technological challenges of using the smart water bottles and interpreting the data from the bottles generated substantial work for the coaches and other staff. The coaches’ jobs also required them to have substantial facility with several study databases to review the adherence data, communicate with other staff, and record their interactions. The work of documentation could distract from their more important efforts of effectively supporting participants. In general, the coaches (and coordinators) needed to acquire a deep understanding of the nuances of the smart bottles including glitches with data transfer and Bluetooth connection. The coaches also noted the specific challenge that some participants spent time in situations, such as a sporting event or a laboratory-based workplace, where bottle use was not allowed. The main solution for coaches was to help participants plan to consume their fluid prescription before or after those situations.

## Discussion

SPS is a promising strategy for helping individuals to systematically develop individualized solutions to improve health behaviors. SPS has been applied in several studies to diet and exercise, as well as diverse other behaviors including medication adherence and adopting habits to alleviate fatigue [17–19, 23, 24]. Here we provide a detailed account of the PUSH trial’s approach to training and assessing intervention fidelity in a multisite design. We also recount challenges for SPS implementation that will likely be faced by other groups developing SPS in trials or real-world practice. These challenges include the difficulty of working effectively with patients who seem unengaged, balancing the need to build rapport with the concrete work of SPS, and maintaining clarity about boundaries in the role of the SPS coach.

Compared with other studies, the PUSH trial design was distinctive because SPS was assigned only to those intervention arm participants who did not succeed in meeting fluid consumption goals in the setting of financial incentives. The rationale for incentives was that financial motivation might enable participants to overcome present bias, the tendency to prioritize behaviors with immediate rewards versus behaviors with future benefits (such as augmenting fluid consumption to lower the risk of a stone on some unknown future date) [30]. However, financial incentives in the PUSH trial (and most other studies) are not customized to the particular needs of participants. The success of financial incentives in PUSH depends upon the capacities of individuals to overcome the diverse personal barriers to drinking fluids. SPS complements the financial incentive intervention by supporting participants to develop feasible solutions. For instance, two recent qualitative studies that focused on improving adherence to statins to reduce cholesterol revealed that for many participants, reduction in LDL-cholesterol was not a salient goal and issues such as limited access to healthy foods posed serious impediments to adopting healthy lifestyles [31, 32]. SPS might address these issues. Further, the “escalation of intervention” design in the PUSH trial was meant to echo the logic of clinical practice, in which participants who do not succeed after initial intervention for a medical condition (such as routine education) will then receive a more intensive intervention.

The challenges faced by health coaches in the PUSH trial may be familiar to health professionals who apply diverse approaches to behavior change such as motivational interviewing. First, some patients may lose interest in the health behavior or become too busy to participate. Even though PUSH participants presumably desired to reduce kidney stone risk, some would not engage with requests to schedule SPS. Only 53% of the intervention arm participants who qualified for SPS went on to complete an initial meeting with a health coach. Coaches had to be persistent (e.g., by sending text messages, emailing, and calling when necessary) and also flexible in scheduling SPS, while recognizing that for some participants, further attempts were not worth the potential cost of being perceived as a nuisance. It is possible that future trials or programs may achieve higher rates of engaging participants if SPS is integrated into in-person clinical visits or if the health coaches are more directly integrated into the clinical care team.

The next challenge identified by coaches — building rapport — takes place after a participant shows that he or she is engaged enough to meet. The PUSH trial’s solutions for having coaches who could skillfully balance rapport-building with staying task-focused was to target hiring individuals with a background in related fields such as social work, and to have the coaches role-play various challenging patient scenarios as part of training. Finally, maintaining clarity with participants about the boundaries of the health coach role was important.

We acknowledge limitations. We did not perform qualitative interviews of study participants to elicit their experience of the interventions [33]. Our aim was instead to evaluate the existing published literature on SPS interventions and provide a detailed, pragmatic account of the implementation of the PUSH interventions. Future studies should collect qualitative data about whether and how SPS helped patients to overcome barriers to behavior change. A second limitation is that some of the issues with SPS implementation in the PUSH trial may not be generalizable to other settings. For instance, the trial relied on a smart water bottle to assess adherence to fluid consumption, and it is likely that some participants were referred to SPS because of underestimation of their actual fluid consumption. While smart devices to monitor adherence have posed challenges in other trials, some SPS interventions may not rely on devices to assess the target health behavior. Third, as shown in Table 1, participants who qualified for SPS had limited racial, ethnic, and socioeconomic diversity. For example, a large majority identified as White and most had private insurance. Finally, we do not report on possible associations between engagement with coaching and outcomes including subsequent kidney stones, adherence to fluid intake recommendations or visiting providers. However, our group will examine for these associations when the parent trial is unblinded and analysed.

In summary, SPS is a promising tool to help patients identify barriers to diverse health behaviors and customize solutions to improve those behaviors. The PUSH trial demonstrated the feasibility of applying SPS to a large controlled clinical prevention trial to help participants with USD overcome their personal barriers to fluid consumption. We provide a detailed account of the training procedures that prepared coaches in the trial to successfully implement SPS across a multisite environment, and challenges identified following SPS implementation.

## Conclusions

SPS supports patient autonomy by helping patients identify goals and strategies that match their personal circumstances. This manuscript provides a detailed account of SPS implementation in the setting of kidney stone prevention and challenges identified following SPS implementation, such as variable patient engagement. This trial also shows how SPS may be adapted to the needs of adolescents and accommodate the involvement of their parents, where necessary. Future studies or clinical programs that develop SPS interventions should anticipate similar problems, which may be addressed through training and ongoing support for coaches in their work. When the PUSH trial is completed, it will be possible to assess the efficacy of the multicomponent intervention, and our team will disseminate results widely.

### Electronic supplementary material

Below is the link to the electronic supplementary material.

Supplementary Material 1

Supplementary Material 2

Supplementary Material 3

Supplementary Material 4

## Data Availability

The datasets used and/or analyzed during the current study are available from the corresponding author on reasonable request.

## Declarations

### Urinary stone disease research network

The following individuals were instrumental in the planning and conduct of the PUSH study at each of the participating institutions:

### Clinical centers

University of Pennsylvania/Children’s Hospital of Philadelphia, Philadelphia, PA: PI: Peter P Reese, MD, PhD; Gregory E. Tasian, MD, MSCE. Co-I: Sandra Amaral, MD, MHS; Janet Audrain-McGovern, PhD. Study Coordinators: Emily Funsten, Brittney Henderson, Kristen Koepsell, Adam Mussell.

University of Texas Southwestern Medical Center, Dallas, TX: PI: Naim M. Maalouf, MD. Co-I: Jodi A. Antonelli, MD; Linda A. Baker, MD; Margaret S. Pearle, MD, PhD; Lakshmi Ananthakrishnan, MD. Study Coordinators: Joyce Obiaro, Cynthia Rangel, Martinez Hill, Madeline Worsham.

University of Washington, Seattle, WA: PI: Jonathan D. Harper, MD; Hunter Wessells, MD; Co-I: Fionnuala Cormack, MD; Mathew Sorensen, MD; Karyn Yonekawa, MD. Study Coordinators: Holly Covert, Tristan Baxter, Elsa Ayala.

Washington University in St. Louis, St. Louis, MO: PI: Alana C. Desai, MD; H. Henry Lai, MD. Co-I: Vincent Mellnick, MD; Douglas Coplen, MD. Study Coordinators: Juanita Taylor, Aleksandra Klim, Deborah Ksiazek.

### Recruiting centers

Cleveland Clinic Foundation, Cleveland, OH: PI: Sri Sivalingam, MD, MSc, FRCSC. Co-I: Katherine Dell, MD; Juan Calle, MD. Study Coordinators: Paige Gotwald, Marina Markovic.

Mayo Clinic Foundation, Rochester, MN: PI: John Lieske, MD; Co-I: Andrew Rule, MD; Stephen Erickson, MD; Aaron Potrezke, MD; Andrea Ferrero, PhD; David Sas, DO. Study Coordinators: Angela Waits, Courtney Lenort.

### Scientific data research center

Duke Clinical Research Institute, Duke University, Durham, NC: PI: Charles D. Scales, Jr, MD, MSHS; Hussein R. Al-Khalidi, PhD. Co-I: Kevin Weinfurt, PhD; Hayden Bosworth, MD. Statistician: Honqiu Yang, PhD. Project Lead: Laura Johnson. Lead CRA: Sharon Settles. CRA: Angela Venetta. Data Manager: Omar Thompson.

*National Institute of Diabetes and Digestive and Kidney Diseases (NIDDK)*.

Project Scientist: Ziya Kirkali, MD. Program Official: Christopher Mullins, PhD.

## Abbreviations

PUSH: Prevention of Urinary Stones with Hydration
SPS: Structured problem solving
USD: Urinary stone disease
