# Supplementary material for: Using structured problem solving to promote fluid consumption in the prevention of urinary stones with hydration (PUSH) trial
Source: BMC Nephrol. 2024 May 28;25:183. doi: 10.1186/s12882-024-03605-y (PMC11134957; doi:10.1186/s12882-024-03605-y)
Supplement: Supplementary file 1 — Supplementary Material 1 [file 12882_2024_3605_MOESM1_ESM.docx]

**Appendix 2: Manual of Procedures: Structured Problem Solving Guide for Coaches to Promote Fluid Intake***

**TABLE OF CONTENTS**

Section 1: Overview of Study Objectives, Rationale, and Design

Section 2: Setting the Stage – How to Build Rapport between the Coach and PUSH participant

Section 3: Structured Problem Solving Guide

Section 4: Structured Problem Solving Worksheet

Section 5: First Structured Problem Solving Session – Step-by-Step

Section 6: Week 1 Check-In – Review and revise implementation plan (SPS Step 6)

Section 7: Monthly Feedback Session

Section 8: SPS Booster 1 – Addressing difficulty with reaching goals

Section 9: SPS Booster 2 – Persistent challenges with achieving goals

Section 10: Communicating with the study team

Section 11: Implementation

Section 12: Fidelity Assessment Worksheet

Section 13: General Troubleshooting

* This MOP was used by coaches in the PUSH study, however, minor changes have been made for manuscript submission.

**Section 1:** **Overview of Study Objectives, Rationale, and Design**

1. Information and video describing what stones are, how they form, and their impact on life;
2. What is structured problem solving and worksheet;
3. Before a coach starts with an actual participant, the coach needs to:
4. Read the training guide
5. Attend the training session in-person or view it online
6. Practice
   1. Practice full session with mock participant
   2. Practice working through SPS worksheet with both a mock participant who is highly adherent and a mock participant who is greatly non-adherent
   3. Practice follow-up calls
7. Review goals and expectations of coach in study – frequency, context and quality of contact with participants
8. Review who is available for support as questions arise and for check-ins on progress

**Section 2: Setting the Stage – How to Build Rapport between the Coach and PUSH participant**

1. Initial contact to schedule 1^st^ SPS Session via participant’s preferred contact method
2. *“Hi [Participant name]. My name is [Coach name]. I am your personalized coach for the PUSH study. You have qualified for coaching because you have not been meeting your fluid goal. In the coaching sessions, we will work together to figure out some of the things that are getting in the way of increasing your fluid intake and to find strategies so that you can reach your goal. Let’s schedule a date and time to meet for our first session. The first session takes about 30 minutes.”*
3. Role of coach is to be empathetic and supportive of participant
   1. Coach encourages participant to reflect on what is difficult for him/her
   2. Reinforce that although many people have difficulties getting in enough fluid intake, the reasons are varied
   3. Coach helps participant work through why it is hard and help identify steps he/she can take to improve
4. Coach creates a safe, judgment-free zone
   1. Coach does not dismiss or judge the experience of the participant
   2. What is and is not shared with healthcare team
   3. mandatory reporting of self-harm and harming others

**Section 3: Structured Problem Solving Guide**

1. Overview of 6 steps
   1. Define the problem
   2. List all possible solutions
   3. Consider each possible solution
   4. Choose the best or most practical solution
   5. Plan how to carry out the best solution
   6. Review progress towards achieving success (achieving fluid goal) and revise implementation plan if needed
2. General core concepts of SPS
   1. The aim is not for the coach to solve everyone’s problems but instead to give people pragmatic solutions and skills so that they can effectively overcome problems and achieve goals for themselves. Self-management is a key goal, with the coach adopting the role of a teacher or guide.
      1. Defining problem in lay language
      2. Encouraging people to seek a wide range of ideas
      3. Carefully considering the practical constraints that must be overcome in successfully applying a solution to increase feasibility of success/meeting goals
3. Specific steps relevant to the PUSH trial
4. Identify root cause(s) of participant’s inability to maintain high fluid intake
   1. Try to identify all the reasons it is difficult for the participant to drink a sufficient amount of fluid each day
      1. Free association by participants
      2. Open ended questions by coaches
      3. Prompts
         1. Work/school barriers
         2. Participant not prioritizing the importance of stone prevention
         3. Sense of abdominal fullness/distaste for additional fluid intake
         4. Lack of thirst
         5. Misperceptions of efficacy of certain fluids (e.g. tea)
         6. Concerns about the safety of water/fluid
         7. Concerns about need to urinate frequently
         8. Lack of ready access to fluid
         9. Lack of ready access to bathrooms
   2. Write down all the reasons in no particular order
5. Identify and list all possible solutions
   1. Open ended: participant driven
   2. Identify and list all possible solutions (no matter how potentially infeasible) for each of the defined barriers
6. Consider each possible solution
   1. Go down the list of possible solutions and assess the main advantages and disadvantages of each one
7. Coach facilitates participant selecting the strongest solution
   1. Considerations
      1. Practicality
      2. Resources
8. Plan how to carry out the strongest solution
   1. List, in small steps, how the participant intends to implement the solution.
   2. Identify resources needed

Adolescent

Parents

School nurse

Teacher

Self-management of urinary frequency

Adult

Partner

Children

Manager

Self-management of urinary frequency

- 1. Identify facilitators and barriers (people and things) and think about how to overcome barriers and optimize facilitators

Suggest having person think about what he/she will do if he/she can’t carry out the solution – who does he/she go to, what’s next to try?

1. Review and revise solutions over subsequent encounters
   - - 1. Review fluid intake data from wireless bottle
       2. Where available, review 24-hour urine collection data
       3. Revise implementation plan as needed

**Section 4: Structured Problem Solving Worksheet**

1. List reasons why it is hard to drink sufficient fluids (My goal fluid intake is ___________.)

______________________________________________________________________________________________________________________________________________________________________________________________________________________________________________________________________________________________________________________________________________________________________________________________________

1. List all possible solutions.

Brainstorm and put down all ideas, even bad ones. List all possible solutions without any evaluation of them at this stage.

______________________________________________________________________________________________________________________________________________________________________________________________________________________________________________________________________________________________________________________________________________________________________________________________________

1. Consider each possible solution

Go down the list of possible solutions and assess the main advantages and disadvantages of each one.

Pros Cons

Solution 1

Solution 2

Solution 3

Solution 4

Solution 5

1. Choose the strongest or most practical solution(s) to drink your goal fluid intake.
2. Plan how to carry out the strongest solution to reach your goal fluid intake.

Problem to overcome:

Resources needed (people and things):

Step 1: _______________________________________________________________________

Step 2: _______________________________________________________________________

Step 3: _______________________________________________________________________

Step 4: _______________________________________________________________________

Step 5: _______________________________________________________________________

Step 6: _______________________________________________________________________

Step 7: _______________________________________________________________________

1. Review and revise implementation plan (within 1 week)

**Section 5:** **First Structured Problem Solving Session – Step-by-Step**

1. Review participant fluid data before session
2. Greet and thank participant/family and reinforce benefits of study
   1. *“Hi [participant name]! My name is [Coach name], I am your PUSH Coach. We are happy to have you as a participant in the PUSH study. Your participation will allow you to take steps to achieve better health and to help doctors understand how maintaining high fluid intake can decrease your chance of kidney stone recurrence or progression. It looks like over the past month you have been having difficulty drinking enough fluid. I want to help you meet your fluid goal!”*
3. Provide overview of coaching
   1. *“Over the next few weeks, we will be meeting to review your progress with your fluid intake and to look at ways to improve that so that you don’t get more kidney stones.”*
4. Provide overview of Session 1
   1. *“Today, we will cover a lot of material. First, we will review why fluid intake is important to decrease stone formation. Then, we will go over how much fluid your healthcare team recommends for you. Next, we will talk about the things that make sticking to your fluid goal difficult. We will work together to find a solution to reach your fluid goal. Finally, we will create a plan that you can use to achieve that solution between now and our next meeting. The goal is to help you increase your fluid intake! As we work through things today, I will take notes. Do you have any questions before we get started?”*
5. Review basic education on stones with participant
   1. Show PowerPoint with video.
   2. *“What questions do you have about the information we just covered? The key takeaway is simple. If you drink a large amount of fluid, you make a large amount of urine, which decreases the formation of stones. But, if you drink a small amount of fluid, you make a small amount of urine and stone formation increases.”*
6. Review fluid goal
   1. *“Great. Let’s get started.”* Review expectations for participant’s fluid goals. “*It looks like your healthcare team/doctor recommends X milliliters or X ounces of EXTRA fluid per day for you to prevent stones. Do you know how much that is? You will need to drink X number of smart water bottle(s) each day.”*
7. Review fluid data for participant from beginning of study
   1. *“I am going to walk through the data that we have collected on your fluid drinking since the start of the study.”*
   2. Coach looks for any trends in days missed: “*It looks like you were doing really well for X weeks/Y days but then tapered off. Do you remember what got in the way?”*
8. Step 1: Identify barriers
   1. *“What would you say are your top 3-4 barriers to drinking more?”*
      1. If barrier identified then have participant reflect back on other barriers: *“What other things get in the way of meeting your fluid goals? It’s common to have lots of things get in the way. Is there anything else you can think of?”*
      2. If he/she says no, refer back to common barriers to stimulate thought process: *“It’s okay that you don’t know why it’s so hard. Lots of people get really busy and don’t give much thought to this. Maybe you find it hard because of no breaks at work* [or something else the participant has not mentioned from the list of common barriers]*”*
         1. Common barriers: having available fluids, available bathrooms, concerns about having to go to the bathroom frequently, concerns about waking up at night to urinate, concerns about the safety of the fluid that is available, thoughts that all fluids may not be helpful, not feeling thirsty, an uncomfortably full stomach, preventing another stone is low on the priority list, other work/school barriers, lack of awareness of how much you drink, lack of awareness of fluid goal, other work/school barriers, unable to access the smart water bottle/app/website.
   2. Positive feedback: *“It’s great that you have a clear idea of the things that make it hard for you to drink water during the day.”*
9. Step 2: Identify and list all possible solutions
   1. ***“****You’ve come up with a great list of things that get in your way. Now, let’s brainstorm and think about ideas of how you might remove these barriers. Don’t worry if the idea might not be realistic, just let your thoughts flow.”*
   2. Walk participant through possible solutions to each barrier: *“Let’s look at barrier <#1>. Off the top of your head, what are 5 things that you can do to remove this barrier?”*
   3. What to do if participant has no ideas: “It *seems like you’re having trouble thinking of solutions. Let’s walk through an average day for you. What time do you get up? When do you first get something to drink? What happens next? So, in what you just described, when do you think you could increase your fluid intake?”*
10. Step 3: Consider each possible solution
    1. *“Now, you’ve come up with a list of possible solutions to drink more fluid. Let’s discuss the pros and cons of each one.”*
       1. Discussing pros and cons of each solution: *“First, you mentioned A. What would be good about choosing A? How do you think you could do A? What might be difficult about doing A? Do you think A is a realistic solution? How confident are you that you can be successful with A?”*
11. Step 4: Choose the strongest solution
    1. *“It is better to have several solutions to a barrier because if one solution does not work, you have another as a back-up. Also, a solution that works on one day or in one situation might not work on another day or in another situation. Now, I need you to rank your solutions from strongest to weakest. The strongest solution is the one that you think would be the best for you to meet your fluid goals.”* Participant chooses solution. “*You have chosen A as your strongest solution. Excellent work!”*
12. Step 5: Plan how to carry out the strongest solution
    1. “*Now, let’s walk through how you will carry out your solution.*” Ask participant to walk through his/her weekday and weekend with solution in mind, i.e. typical day – but keep in mind any changes in routine. “*What problems do you think might come up? What resources (resources can be people or things) will you need to be successful*? *Who can you ask for help? How can I help? (e.g. providing letter to teacher/manager)*” If participant doesn’t say anything, ask specifically about people he/she interacts with during the day, people who provide support, or tangible things (setting alarm, sticky note to remember water bottle, reminder to wash bottle, etc.).
13. Plan implementation
    1. *“Sounds like you’re coming up with a great plan for action. How will you know you’re making progress? If you’re not making progress, what will you do? Let’s define a date that you will start doing your plan. When would you like to begin?”*
14. End session
    1. *“Thanks for all the hard work you put in with me today to increase your fluid intake. Let me summarize what we talked about today. Your goal is to drink X amount of fluid. Your plan to meet your goal is to address barrier X by solution A. You plan is the following: [list steps]. Your plan sounds great and will help you decrease your risk for painful kidney stones. I will send you a copy of what we discussed today. Let’s schedule a time on [1 week date] to check in and see how things are going.”*
    2. Schedule date, time, and format (voice call, Skype, screen share, etc.) for week 1 check-in.

**Section 6: Week 1 Check-In – Review and revise implementation plan (SPS Step 6)**

1. Review participant fluid data before call
2. Greet family/participant and provide overview of session
   1. *“Hi [Participant name]. This is [Coach name], your PUSH Coach. I am just calling to check in. During our last session, you did a great job at identifying many solutions to help you achieve a higher fluid intake. You chose the best option which was A. You decided to start doing A on [date], did you stick to your plan? How successful were you in completing your plan?”*
3. Address participant concerns and answer any questions. Use reflective listening to summarize participant thoughts
   1. “What I hear you saying is X, it sounds like Y has been difficult this week. It sounds like X was frustrating, tell me more about that, etc.”
4. If the implementation of A is going well, advise participant to continue forward
   1. *“It sounds like you have been doing a great job drinking more fluids. It can be difficult to achieve a goal in such a short period of time and you have succeeded! Awesome work! What can you do in the upcoming weeks to make sure that you keep making progress?”*
5. If the implementation of plan A is not going well, help participant devise a new plan
   1. *“Why do you think you were not able to carry out A? The steps that you outlined were W, X, Y, Z. Have you been able to do these? Is there one that you may have forgotten? Let’s talk about how you can modify your plan to achieve your fluid goal.”*
6. Plan implementation
   1. *“How can you carry out that plan? What do you need? Do you need help? Who can you ask for help? How can I help? When would you like to start? How will you know if you are making progress? If you are not making progress, what will you do?”*
7. End session
   1. *“Thanks for taking with me today about your fluid intake. Your plan for the upcoming weeks sounds great and will help you decrease your risk for painful kidney stones. I will check in with you in a few weeks to see how things are going. I will also send you a copy of what we discussed today. Let’s schedule a time on [1 month date] to check in and see how things are going.”*
   2. Schedule date, time, and format (voice call, Skype, screen share, etc.) for monthly feedback session

**Section 7: Monthly Feedback Session**

1. Review participant fluid data before call
2. Greet family/participant and provide overview of session
   1. *“Hi [Participant name]. The purpose of this session is to see how things are going since our last session on [date] and if need be modify the plan that we worked on together during the last session.”*
3. Review data
   1. *“Let’s get started. How have things been going? Have you noticed any patterns like days of the week or times when it is harder to drink more fluids?”*
      1. If participant says yes: *“Let’s look at your report and see if that matches what you are thinking.”*
      2. If participant says no: *“Okay, sometimes it is hard to reflect back on our day to day activities. Let’s look at your fluid intake report and see what days are not working. It looks like on these days [state specific days], you are not meeting your goal. What can you do to drink more fluids on those days? During our last session, we talked about taking these steps [list steps] to help you drink more fluids. What steps can you change?”*
4. If participant needs a new solution, bring up list of solutions from first SPS session
   1. *“From our first meeting, you had solutions A, B, and C. You have been working on A, what about considering B and C. Let’s talk about those. Which one sounds good to you? How feasible is that solution? Have you thought of any new solutions?”*
5. Make plan
   1. *“Drinking fluids is very important to decrease your risk of kidney stones. You know yourself and your schedule the best. Let’s make a plan of how you can drink more fluid on the days that you identified. Oftentimes having a plan can make a task seem not as big and every step that you achieve in that plan is another step toward success.”*
6. Plan implementation
   1. *“What steps can you take to carry out that plan? What are some resources that you may need or people who can help you? How can I help? How will you know if you are making progress? If you are not making progress, what will you do? When would you like to start?”* Decide on start date with participant.
7. End session
   1. *“Thanks for all the hard work you put in with me today to increase your fluid intake. Your plan sounds great and will help you decrease your risk for painful kidney stones. I will check in with you in a few weeks to see how things are going. I will also send you a copy of what we discussed today. Let’s schedule a time on [1 month date] to check in and see how you are doing.”*
   2. Schedule date, time, and format (voice call, Skype, screen share, etc.) for next monthly feedback session

**Section 8: SPS Booster 1 – Addressing difficulty with reaching goals**

1. Review participant fluid data before session
2. Greet family/participant
   1. *“Hi [Participant name”]. This is [Coach name], your PUSH coach. It looks like you have been having difficulty drinking enough fluid. Today we are going to problem-solve how you can reach your daily fluid goal.”*
3. Review basic education
   1. *“As you may remember from our very first session, drinking fluids is very important to prevent kidney stones. Do you remember why? It’s simple. If you drink a large amount of fluid, you make a large amount of urine which decreases the formation of stones. If you drink a small amount of fluid, you make a small amount of urine and stone formation increases. As your coach, I want to help you meet your fluid goal so you do not develop another painful kidney stone.”*
4. Review goal and data
   1. *“Let’s get started with today’s session. Do you remember your fluid goal? Your fluid goal is X milliliters or X ounces of EXTRA fluid per day for you to prevent stones. You will need to drink X number of smart water bottle(s) each day.”*
   2. Review fluid data
      1. *“I took a look at your fluid data before our call and it looks like you were doing really well for X weeks/Y days but then tapered off. Do you remember what got in the way? We all have times of the day when we drink more fluid and times where we drink less. How did those days that you met your hydration goal differ from those days that you did not? Let’s consider hydration troughs and peaks to help you think about when you are drinking fluid and when you are not drinking fluid.”*
      2. Hydration troughs: *“What part of the day do you drink less? What makes it difficult to drink more fluid during those times? The things that make it difficult to drink more fluid are called barriers.”*
      3. Hydration peaks: *“What parts of the day do you drink more? What makes it easier to drink more fluid during those times? The things that make it easier to drink more fluid are called facilitators. How might you use your facilitators to drink even more fluid during those parts of the day?”*
5. Step 1: Identify barriers
   1. *“What would you say are your top 3-4 barriers to drinking more? What other things get in the way of you meeting your fluid goals? It’s common to have lots of things get in the way. Is there anything else you can think of?”*
      1. If he/she says no, refer back to common barriers to stimulate thought process: *“It’s okay that you don’t know why it’s so hard. Lots of people get really busy and don’t give much thought to his. Some people find it hard because of no breaks at work* [or something else the participant has not mentioned from list of common barriers]*. Try to consider when you are able to drink fluid, those hydration peaks. What makes it easy drink during those time periods?”*
      2. Common barriers: having available fluids, available bathrooms, concerns about having to go to the bathroom frequently, concerns about waking up at night to urinate, concerns about the safety of the fluid that is available, thoughts that all fluids may not be helpful, not feeling thirsty, an uncomfortably full stomach, preventing another stone is down on the priority list, other work/school barriers, lack of awareness of how much you drink, lack of awareness of goals, other work/school barriers, unable to access the smart water bottle/app/website.
   2. Positive feedback: *“It’s great that you have a clear idea of the things that make it hard for you to drink water during the day.”*
6. Step 2: Identify and list all possible solutions
   1. ***“****You’ve come up with a great list of things that get in your way. Now, let’s brainstorm and think about ideas of how you might remove these barriers. Don’t worry if the idea might not be realistic, just let your thoughts flow.”*
   2. Walk participant through possible solutions to each barrier
      1. *“Let’s look at barrier <#1>. Off the top of your head, what are 5 things that you can do to remove this barrier?”*
   3. What to do if participant has no ideas
      1. “It *seems like you’re having trouble thinking of solutions. Let’s walk through an average day for you. What time do you get up? When do you first get something to drink? What happens next? So, in what you just described, when do you think you could increase your fluid intake?”*
7. Step 3: Consider each possible solution
   1. *“Now, you’ve come up with a couple of possible solutions to drink more fluid. Let’s cross off those that are just not feasible. Now, from the remaining solutions, let’s discuss the pros and cons of each one.”*
8. Discussing pros and cons of each solution: *“First, you mentioned A. What would be good about choosing A? How do you think you could do A? What might be hard about doing A? Do you think A is a realistic solution? How confident are you that you can be successful with A?”*
9. Step 4: Choose the strongest solution
   1. *“It is better to have several solutions to a barrier because if one solution does not work, you have another as a back-up. Also, a solution that works on one day or in one situation might not work on another day or in another situation. Now, I need you to rank your solutions from strongest to weakest. The strongest solution is the one that you think would be the best for you to meet your fluid goals.”* Participant chooses solution. “*You have chosen A as your strongest solution. Excellent work!”*
10. Step 5: Plan how to carry out the strongest solution
    1. *“Now, let’s walk through how you will carry out your solution.”* Ask participant to walk through his/her weekday and weekend with solution in mind, i.e. typical day – but keep in mind any changes in routine. “*What problems do you think might come up? What resources (resources can be people or things) will you need to be successful? Who can you ask for help? How can I help?”* If participant doesn’t say anything, ask specifically about people he/she interacts with during the day, people who provide support, or tangible things (setting alarm, sticky note to remember water bottle, reminder to wash bottle, etc.). “*What difficult tasks do you think you will encounter and how will you fix them? How will you tell if you have succeeded?”*
11. Plan implementation
    1. *“Sounds like you’re coming up with a great plan for action. How will you know you’re making progress? If you’re not making progress, what will you do? Let’s define a date that you will start doing your plan. When would you like to begin?”*
12. End session
    1. *“Thanks for all the hard work you put in with me today to increase your fluid intake. Let me summarize the plan that you came up to meet your fluid goal. Your goal is to drink X amount of fluid. Your plan to meet your goal is to address barrier X by solution A. Your plan is the following: [list steps]. Your plan sounds great and will help you decrease your risk for painful kidney stones. I will send you a copy of what we discussed today. Let’s schedule a time on [1 week date] to check in and see how things are going.”*
    2. Schedule date, time, and format (voice call, Skype, screen share, etc.) for monthly feedback session

**Section 9: SPS Booster 2 – Persistent challenges with achieving goals**

1. Review participant fluid data before session
2. Greet family/participant
   1. *“Hi [Participant name”]. This is [Coach name], your PUSH coach. It looks like you have been having difficulty drinking enough fluid. Today we are going to problem-solve how you can reach your daily fluid goal.”*
3. Review basic education
   1. *“As you may remember from our very first session, drinking fluids is very important to prevent kidney stones. Do you remember why? It’s simple. If you drink a large amount of fluid, you make a large amount of urine which decreases the formation of stones. If you drink a small amount of fluid, you make a small amount of urine and stone formation increases. As your coach, I want to help you meet your fluid goal so you do not develop another painful kidney stone.”*
4. Review goal and fluid data
   1. *“Let’s get started with today’s session. Do you remember your fluid goal? Your fluid goal is X milliliters or X ounces of EXTRA fluid per day for you to prevent stones. You will need to drink X number of smart water bottle(s) each day.”*
   2. Review data
      1. *“I took a look at your fluid data before our call and it looks like you were doing really well for X weeks/Y days but then tapered off. Do you remember what got in the way? We all have times of the day when we drink more fluid and times where we drink less. How did those days that you met your hydration goal differ from those days that you did not? Let’s consider hydration troughs and peaks to help you think about when you are drinking fluid and when you are not drinking fluid.”*
      2. Hydration troughs: *“What part of the day do you drink less? What makes it difficult to drink more fluid during those times? The things that make it difficult to drink more fluid are called barriers.”*
      3. Hydration peaks: *“What parts of the day do you drink more? What makes it easier to drink more fluid during those times? The things that make it easier to drink more fluid are called facilitators. How might you use your facilitators to drink even more fluid during those parts of the day?”*
5. Step 1: Identify barriers
   1. *“What would you say are your top 3-4 barriers to drinking more? What other things get in the way of you meeting your fluid goals? It’s common to have lots of things get in the way. Is there anything else you can think of?”*
      1. If he/she says no, refer back to common barriers to stimulate thought process: *“It’s okay that you don’t know why it’s so hard. Lots of people get really busy and don’t give much thought to his. Some people find it hard because of no breaks at work* [or something else the participant has not mentioned from list of common barriers]*. Try to consider when you are able to drink fluid, those hydration peaks. What makes it easy drink during those time periods?”*
      2. Common barriers: having available fluids, available bathrooms, concerns about having to go to the bathroom frequently, concerns about the safety of the fluid that is available, thoughts that all fluids may not be helpful, not feeling thirsty, an uncomfortably full stomach, preventing another stone is down on the priority list, other work/school barriers, lack of awareness of how much you drink, lack of awareness of goals, other work/school barriers, unable to access the smart water bottle/app/website.
   2. Positive feedback: *“It’s great that you have a clear idea of the things that make it hard for you to drink water during the day.”*
6. Step 2: Identify and list all possible solutions
   1. ***“****You’ve come up with a great list of things that get in your way. Now, let’s brainstorm and think about ideas of how you might remove these barriers. Don’t worry if the idea might not be realistic, just let your thoughts flow.”*
   2. Walk participant through possible solutions to each barrier
      1. *“Let’s look at barrier <#1>. Off the top of your head, what are 5 things that you can do to remove this barrier?”*
   3. What to do if participant has no ideas
      1. “It *seems like you’re having trouble thinking of solutions. Let’s walk through an average day for you. What time do you get up? When do you first get something to drink? What happens next? So, in what you just described, when do you think you could increase your fluid intake?”*
7. Step 3: Consider each possible solution
   1. *“Now, you’ve come up with a couple of possible solutions to drink more fluid. Let’s cross off those that are just not feasible. Now, from the remaining solutions, let’s discuss the pros and cons of each one.”*
8. Discussing pros and cons of each solution: *“First, you mentioned A. What would be good about choosing A? How do you think you could do A? What might be hard about doing A? Do you think A is a realistic solution? How confident are you that you can be successful with A?”*
9. Step 4: Choose the strongest solution
   1. *“It is better to have several solutions to a barrier because if one solution does not work, you have another as a back-up. Also, a solution that works on one day or in one situation might not work on another day or in another situation. Now, I need you to rank your solutions from strongest to weakest. The strongest solution is the one that you think would be the best for you to meet your fluid goals.”* Participant chooses solution. “*You have chosen A as your strongest solution. Excellent work!”*
10. Step 5: Plan how to carry out the strongest solution
    1. *“Now, let’s walk through how you will carry out your solution.”* Ask participant to walk through his/her weekday and weekend with solution in mind, i.e. typical day – but keep in mind any changes in routine. “*What problems do you think might come up? What resources (resources can be people or things) will you need to be successful? Who can you ask for help? How can I help?”* If participant doesn’t say anything, ask specifically about people he/she interacts with during the day, people who provide support, or tangible things (setting alarm, sticky note to remember water bottle, reminder to wash bottle, etc.). “*What difficult tasks do you think you will encounter and how will you fix them? How will you tell if you have succeeded?”*
11. Plan implementation
    1. *“Sounds like you’re coming up with a great plan for action. How will you know you’re making progress? If you’re not making progress, what will you do? Let’s define a date that you will start doing your plan. When would you like to begin?”*
12. End session
    1. *“Thanks for all the hard work you put in with me today to increase your fluid intake. Let me summarize the plan that you came up to meet your fluid goal. Your goal is to drink X amount of fluid. Your plan to meet your goal is to address barrier X by solution A. Your plan is the following: [list steps]. Your plan sounds great and will help you decrease your risk for painful kidney stones. I will send you a copy of what we discussed today. Let’s schedule a time on [1 week date] to check in and see how things are going.”*
    2. Schedule date, time, and format (voice call, Skype, screen share, etc.) for monthly feedback session

**SDRC to review:**

**Section 10: Communicating with the study team**

1. Who is point of contact if coach has questions
2. What does coach need to record, when, how often do coach need to submit this information?
3. Mandatory reporting

**Section 11: Implementation**

1. Review protocol with focus on what coach does, where are touch points with participants
2. Communication tracking log

**Section 12: Fidelity Assessment Worksheet**

1. How does coach know if he/she is doing a good job

**Section 13: General Troubleshooting**

1. Participant does not respond to contact from Coach
   1. First SPS Session, Monthly Feedback Session, SPS Booster 1, and SPS Booster 2
      1. Coach contacts the participant by his/her preferred method one time daily for three days
      2. If after 3 days, the participant does not respond, the coach notifies the study coordinator
   2. Week 1 Check-In
      1. Coach contacts the participant by his/her preferred method one time on the next business day
      2. If the participant does not respond, the coach contacts the participant at the end of week 4 for the monthly feedback session
2. Participant suicide ideation
3. Coach coping mechanisms
   1. Monthly calls with other coaches
4. Parent/Partner Participation
   1. Ways to address parent/partner involvement in SPS sessions
      1. *“Hello [Parent/Partner name]. The purpose of these coaching sessions is to focus on [Participant name] and to help him/her develop the strategies needed to meet the fluid goal. We appreciate your enthusiasm and willingness to help [participant name]. The best way to continue forward is if you listen to the introductory portion of the session and then come back for the closing remarks and wrap-up at the end. [Participant name], how does that plan sound?”*
      2. If parent/partner insists on remaining in session, keep engaging participant
         1. *“It is important that we know what [Participant name] thinks. [Participant name] is actively participating in this study so we really need to hear from her/her. Let’s see what he/she has to say. [Participant name], what do you think? We value your opinion, what are your thoughts?”*
5. Coach-Participant Communication Outside of Sessions
   1. Participant initiates contact
      1. Issue with bottle
         1. If simple: help participant
         2. If complex: refer participant to study coordinator
      2. Problem with plan implementation
         1. Instruct participant to reflect on why things are not going well
            1. *“What I hear you saying is that things have not been going well due to X. It sounds like you are frustrated and disappointed. Is that correct? What I would like you do to over the next few days is really try to understand why you are having difficulty. Think about what things are getting in the way of your progress. In our first session, we went through the step by step process of identifying challenges and then coming up with solutions and then a plan. Over the next few days, try a similar process to reflect on the challenges that you may be experiencing. Then, when we talk again we can have a substantive conversation. Do you have any questions?”*
      3. Casual conversation
         1. Re-focus participant back to hydration and fluid goal
