# Supplementary material for: Using structured problem solving to promote fluid consumption in the prevention of urinary stones with hydration (PUSH) trial
Source: BMC Nephrol. 2024 May 28;25:183. doi: 10.1186/s12882-024-03605-y (PMC11134957; doi:10.1186/s12882-024-03605-y)
Supplement: Supplementary file 2 — Supplementary Material 2 [file 12882_2024_3605_MOESM2_ESM.docx]

**Appendix 3:** **SPS coach training materials — hypothetical phenotypes of participants with urinary stone disease**

Adult

1. Teacher
   1. Clara Brooke is a 40-year-old third-grade teacher at her local public school.
   2. During the week, she arrives to school very early and leaves in the evening because she is always assigned to bus duty. On the weekends, she tutors and develops lesson plans for the coming week.
   3. She understands why she needs to increase her fluid intake but does not know how to incorporate drinking into her busy schedule. Clara has been manually adding fluids.
2. Orthopedic Surgeon
   1. Timothy Clancy, MD is a 50-year-old orthopedic surgeon.
   2. He is in the OR 3 days per week and on call 2 times per week.
   3. He thinks he knows why it is important to drink, but as he says, “It is impossible for me to increase my fluid intake!”
3. Uber Driver
   1. Paul Spence is 52 years old and married (to his high school sweetheart Ashley).
   2. A few months ago, he was laid off from his stressful job in tech and thought that becoming an Uber driver seemed like a nice change of pace.
   3. He does not understand the importance of drinking more fluid.
4. Widower
   1. William O’Toole is an 85-year-old retiree.
   2. He also has benign prostatic hyperplasia.
   3. A few months ago, his wife of 45 years, Margie, passed away from cancer. He does not have children, and he does not care about his kidney health and drinking more fluid.
5. Grandmother
   1. Lisa Simpson is a 70-year-old mother of 3 and grandmother of 2.
   2. She suffers from urge incontinence.
   3. She understands why it is important to drink but just does not know how she can realistically drink more.
6. Retiree
   1. Bob MacMullin is a 65-year-old businessman who recently retired and is looking forward to gardening and hiking.
   2. He is a recurrent stone former and would like to start drinking more fluid.
   3. Bob has a supportive family and is a willing participant in coaching.
7. Truck Driver
   1. Mark Peabody is a 53-year-old truck driver.
   2. He has had several kidney stones, and his wife, Susan, is very concerned about his lack of hydration.
   3. Mark’s travel schedule (e.g., hours driving, inaccessibility to a clean bathroom) makes staying hydrated quite difficult.
8. The Millennial
   1. Lyndsay Nichols is a 32-year-old consultant at Amazon.
   2. She just got promoted and purchased a condo in downtown Seattle.
   3. She is not motivated to drink more, does not feel thirsty, and is not very effusive.
9. Construction Worker
   1. Thomas Payne is a 45-year-old construction worker and consultant.
   2. He recently had a kidney stone and was traumatized by the experience.
   3. Thomas travels during the day between job sites and has many important meetings in the office. He thinks the bottle is a nuisance to take with him, it does not look professional, and would feel awkward declining another beverage offered to him by the meeting host.
10. Persistent Bottle Issues
    1. Janice Barnes is a 62-year-old retired executive and currently teaches yoga.
    2. She has had a history of stones since the age of 40.
    3. Janice is not tech savvy and immensely dislikes the bottle due to the plastic taste and bottle/data issues. She has given up using the bottle.

Adolescent

1. High School Sophomore
   1. Courtney is a 15-year-old student at Milton Academy.
   2. She recently transferred there from her local public school and is trying to fit in among her peers.
   3. Her mom and urologist keep telling her that it is important to drink more fluids, but she does not care.
2. Athlete
   1. Grant is a high school junior.
   2. He is very involved in sports (soccer, track, and lacrosse), and his schedule is packed with school, sports, and social events.
   3. Grant is engaged in coaching and is motivated to meet his fluid goal but needs some strategies to incorporate drinking into his busy schedule.
3. New Start
   1. Lizzy is a 14-year-old middle school student.
   2. She has had three kidney stones, and her mother is very worried about her hydration so will be joining the coaching sessions.
   3. Lizzy is an excellent student and is excited to start high school, but she’s not too keen on coaching and talking with the coach about her health information.
4. Off to College
   1. Katie is a high school senior.
   2. She had surgery in the last year to remove a stone and was not very affected by the experience.
   3. Katie is involved in four student clubs and is super busy studying for the SAT and embarking on the college process (e.g., school tours, information sessions). She is very school oriented and thinks that the study as a whole is a waste of her time and her mom pushed her to enroll.
