# Supplementary material for: Using structured problem solving to promote fluid consumption in the prevention of urinary stones with hydration (PUSH) trial
Source: BMC Nephrol. 2024 May 28;25:183. doi: 10.1186/s12882-024-03605-y (PMC11134957; doi:10.1186/s12882-024-03605-y)
Supplement: Supplementary file 3 — Supplementary Material 3 [file 12882_2024_3605_MOESM3_ESM.docx]

**Appendix 4:** **Structured problem solving fidelity assessment**

The purpose of the fidelity assessment activities are to ensure consistency in how the structured problem solving coaching is conducted across institutions and between participants. There are key elements that should be covered in the SPS coaching sessions; the Fidelity Assessor will be checking to make sure these elements were addressed and will be providing constructive feedback to the coaches.

## Fidelity Assessor Training

Fidelity Assessors are trained by completing the following activities:

- Reviewing the study protocol
- Viewing the 3 recordings from the SPS coach training
- Reviewing the PUSH Health Coach Manual of Procedures and worksheets used by coaches
- Reviewing the participant-facing videos employed by the SPS coaches:
  1. Fluid Intake and Kidney Stones Tutorial
  2. Approach to Behavior Change Video
- Reviewing Fidelity Assessment worksheets

## Initial Fidelity Assessment Schedule

The Fidelity Assessor will begin by reviewing the following SPS coaching recordings for **each coach**:

| Session Type | Adult | Adolescent | Purpose |
| --- | --- | --- | --- |
| First SPS Session | 3 randomly selected sessions | 3 randomly selected sessions | To familiarize the Fidelity Assessor with the coach’s performance of these sessions over time. |
| First SPS Session Week 1 Check-in | 3 randomly selected sessions | 3 randomly selected sessions | To familiarize the Fidelity Assessor with the coach’s performance of these sessions over time. |
| First SPS Session Month 1 and Month 2 Check-In | 3 randomly selected sessions | 3 randomly selected sessions | To familiarize the Fidelity Assessor with the coach’s performance of these sessions over time. |
| Booster 1 Session | 3 randomly selected sessions | 3 randomly selected sessions | To familiarize the Fidelity Assessor with the coach’s performance of these sessions over time. |
| Booster 2 Session | 3 randomly selected sessions | 3 randomly selected sessions | To familiarize the Fidelity Assessor with the coach’s performance of these sessions over time. |

## Performing Initial Fidelity Assessments

**1) The Fidelity Assessor Completes Review of Coaching (described above)**

**2) The Coach Completes the Self-Assessment based on a session**

**3) Feedback Discussion Between Coach and Fidelity Assessor and the SPS leader**

## Continued Fidelity Assessment Schedule and Performing Continued Assessments

After the initial fidelity assessments have been completed for a coach, the Fidelity Assessor will continue by reviewing the following SPS coaching recordings for **each coach**:

| Session Type | Adult | Adolescent | Purpose |
| --- | --- | --- | --- |
| First SPS Session | One per quarter, randomly selected | One per quarter, randomly selected | To assess coach’s continued performance of these sessions. |
| First SPS Session Week 1 Check-in | One per quarter, randomly selected | One per quarter, randomly selected | To assess coach’s continued performance of these sessions. |
| First SPS Session Month 1 Check-In | One per quarter, randomly selected | One per quarter, randomly selected | To assess coach’s continued performance of these sessions. |
| Booster 1 Session | One per quarter, randomly selected | One per quarter, randomly selected | To assess coach’s continued performance of these sessions. |
| Booster 2 Session | One per quarter, randomly selected | One per quarter, randomly selected | To assess coach’s continued performance of these sessions. |

The Fidelity Assessor, the SPS Lead, and the coach will schedule a time to meet and review the Worksheets and discuss any follow-up actions needed.

## Feedback Escalation

If at any time the Fidelity Assessor has concerns about the performance of a coach, the SPS Lead and the Project Leader should be notified. A meeting will be convened with the Fidelity Assessor to discuss the concerns and determine the appropriate response. Feedback will be shared with the coach in real-time, to allow the coach the opportunity to respond and make changes to how she/he performs the coaching sessions.

In the event of prolonged or repeated issues, the levels of escalation are as follows:

| Level | Action |
| --- | --- |
| 1 | Retraining of the SPS coach and documentation of that retraining |
| 2 | Notification of the PI(s) for the coach’s sites |
| 3 | Notification of Executive Committee (if applicable) |
| 4 | Replacement of the coach |
