# Supplementary material for: Using structured problem solving to promote fluid consumption in the prevention of urinary stones with hydration (PUSH) trial
Source: BMC Nephrol. 2024 May 28;25:183. doi: 10.1186/s12882-024-03605-y (PMC11134957; doi:10.1186/s12882-024-03605-y)
Supplement: Supplementary file 4 — Supplementary Material 4 [file 12882_2024_3605_MOESM4_ESM.docx]

**Appendix 1:** **Overview of the approach to training in structured problem solving for the PUSH trial**

Coaching training included discussion of the limits of the coaching role. An effective coach was described as someone who acted as a guide, advisor, instructor, listener, or resource related to drinking fluids and health behaviors to avoid kidney stones. Training emphasized that a coach was not a therapist, parent, expert, or disciplinarian. Key coaching behaviors were described as building rapport with the participant and active listening, such as summarizing and rephrasing information back to the participant. Coaches were encouraged to facilitate and to guide the participant through the SPS process but not to tell the participant what to do or to develop solutions for the participants.

The coaches were provided with an SPS checklist to ensure consistency in implementation. Coaches were instructed to first review the participant’s specific fluid intake data (recorded from the smart water bottle) with the participant to ascertain whether low fluid intake was due to true low ingestion versus nonuse of the bottle or technical device issues. If true low intake was identified, the coach sought to elicit the participant’s specific barriers to greater intake. For example, one participant might have employment-related barriers or lack of access to a bathroom (e.g., Uber driver), while another might be involved in demanding childcare and suffer from urge urinary incontinence, and a third might have recently suffered a personal setback and be too preoccupied with grief to focus on positive health changes. Next, once the coach elicited the participant’s specific barriers to greater fluid intake, the coach and participant were instructed to work systematically on potential solutions and to examine each for feasibility.

During training, coaches were provided with certificates documenting progress through specific milestones, such as having completed sufficient training to do SPS initial or booster meetings.

Training also leveraged shared knowledge and peer support for the coaches. This support structure included monthly conference calls with all coaches, where discussion of successful practices and challenges with elements of the protocol or with specific participants took place; minutes were recorded for subsequent review. Training sessions were video-recorded for use by coaches hired later in the trial.

**References***

Nezu AM, Nezu CM, D’Zurilla TJ: **Problem-Solving Therapy**, 1 edn. New York: Springer Publishing Company.

Resnicow K, McMaster F: **Motivational Interviewing: moving from why to how with autonomy support**. *Int J Behav Nutr Phys Act* 2012, **9**:19

* Please see main manuscript for additional references related to structured problem-solving.
